# Supplementary material for: Single-cell and spatial detection of senescent cells using DeepScence
Source: bioRxiv. 2025 Apr 23:2023.11.21.568150. Originally published 2023 Nov 21. Preprint. [Version 3] doi: 10.1101/2023.11.21.568150 (PMC10690237; doi:10.1101/2023.11.21.568150)
Supplement: Supplement 1 [file media-1.pdf]

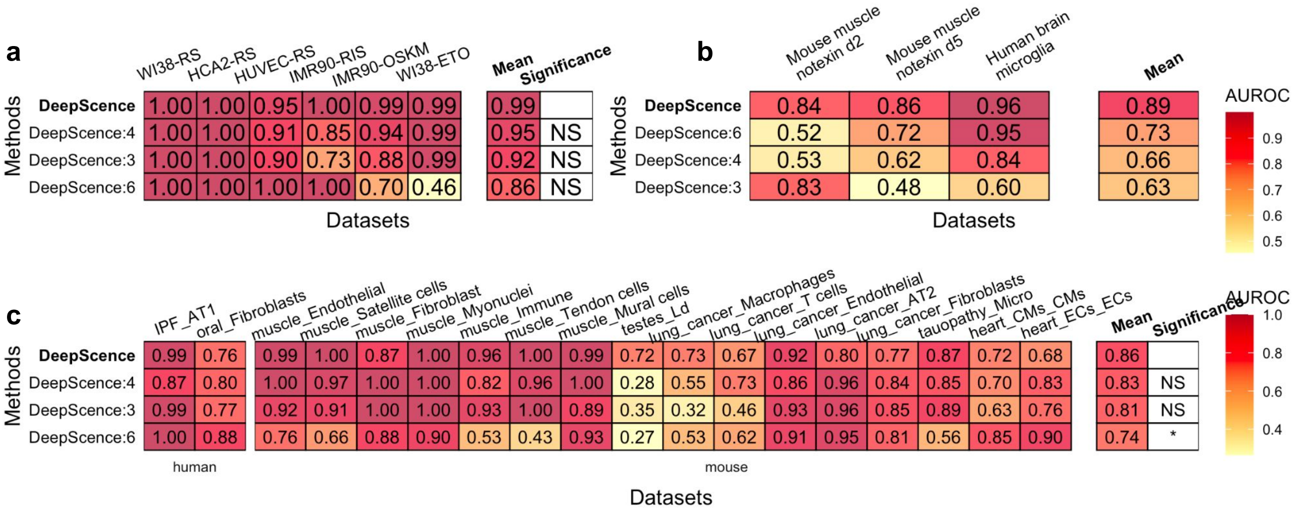

Supplementary Figure 1. AUROCs for DeepScence using different thresholds for constructing CoreScence across *in vitro* scRNA-seq datasets (**a**), ST datasets (**b**), and *in vivo* scRNA-seq datasets (**c**). Methods are ordered in decreasing order of average AUROC. Paired t-tests were conducted to compare the performance between default DeepScence and its variants. "*" indicates 0.01 < p-value < 0.05, and "NS" indicates not significant (p-value > 0.05).

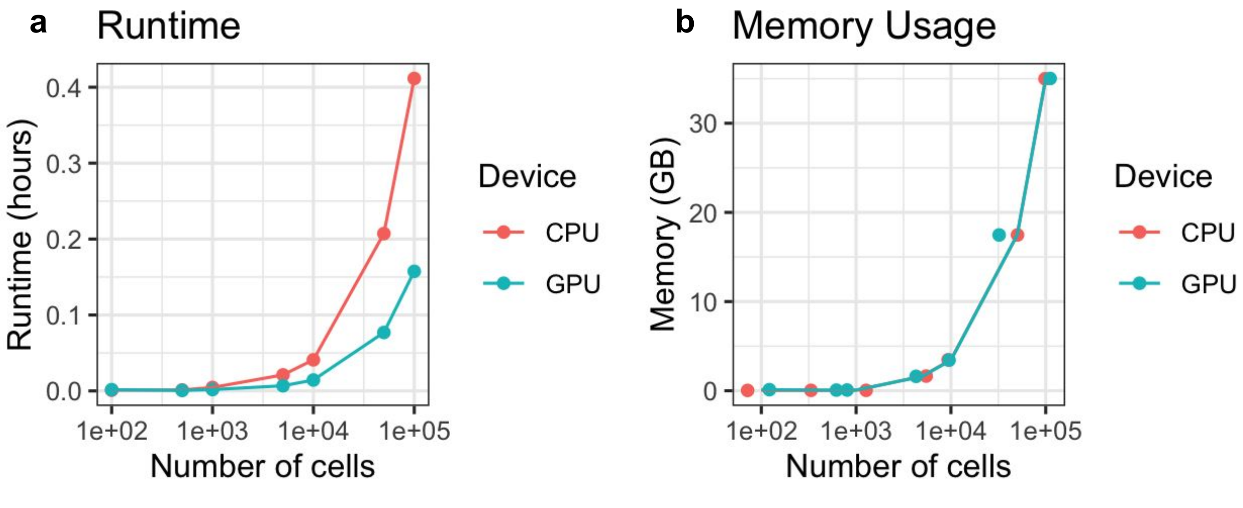

Supplementary Figure 2. Runtime (**a**) and peak memory usage (**b**) for executing DeepScence with different numbers of cells in the input data.

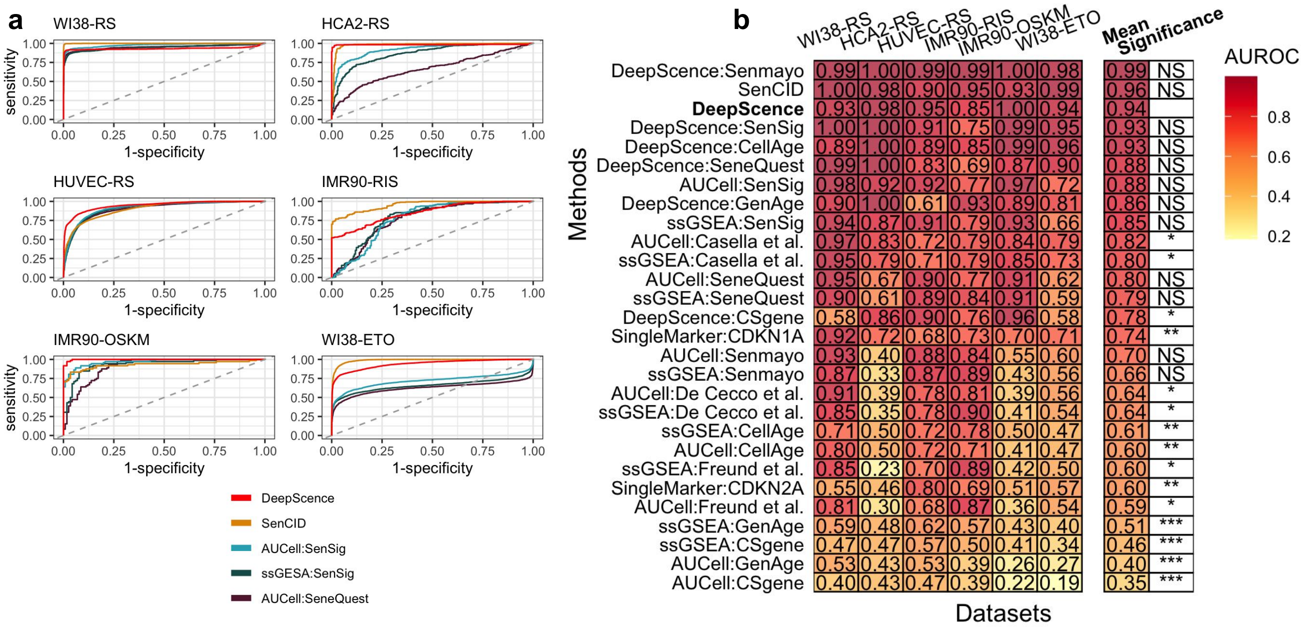

Supplementary Figure 3. a, AUC curves for the top 5 performing methods among *in vitro* datasets under standard data preprocessing pipeline. **b,** AUROCs for all methods among *in vitro* datasets under standard data preprocessing pipeline. Methods are ordered in decreasing order by average AUROCs. Paired t-test was conducted to compare the performance between DeepScence and each of the other methods. “***” indicates p-value < 0.001, "**" indicates 0.001 < p-value < 0.01, "*" indicates 0.01 < p-value < 0.05, and "NS" indicates not significant (p-value > 0.05).

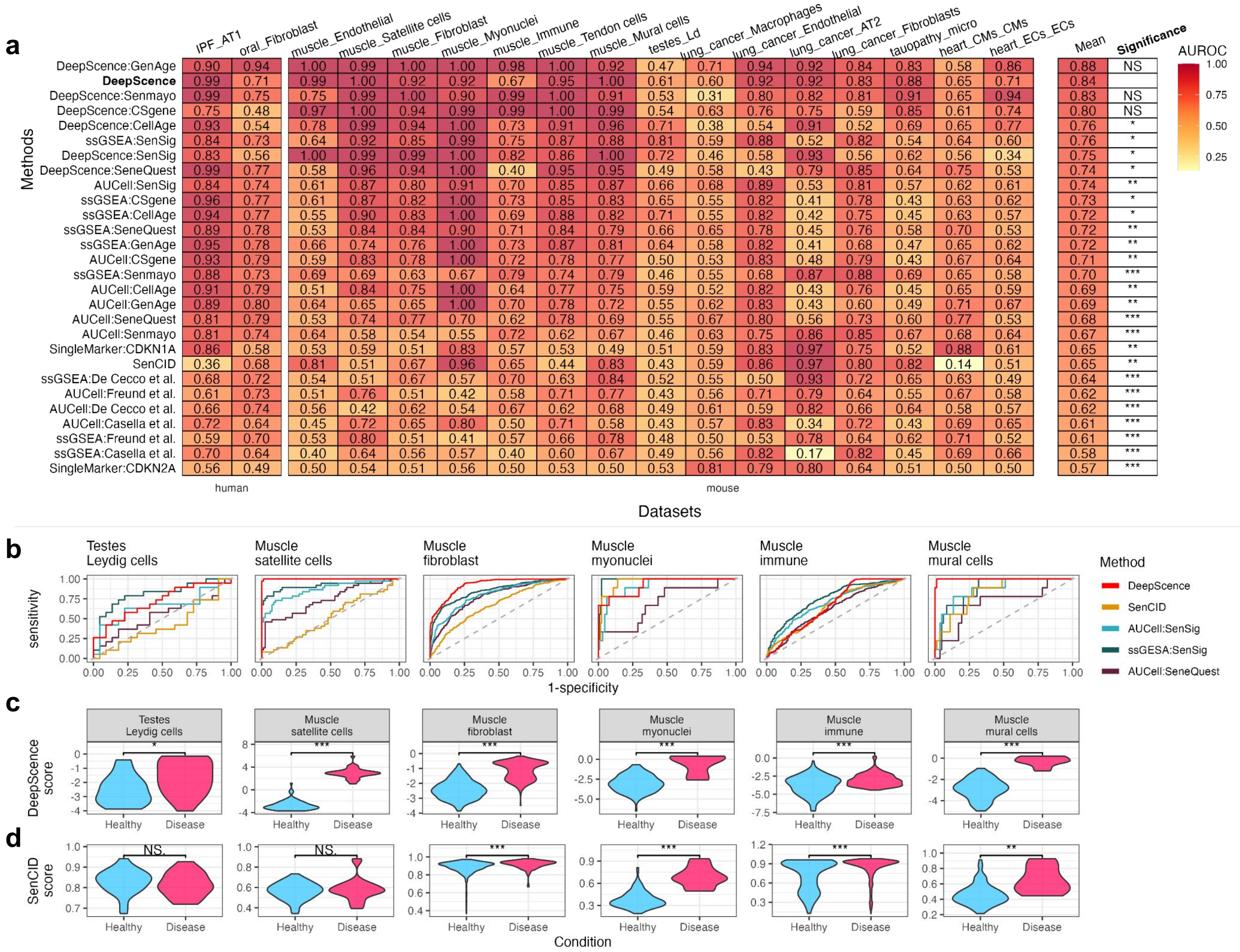

Supplementary Figure 4. a, AUROCs for all methods across *in vivo* datasets under standard preprocessing pipeline. Methods are ordered in decreasing order by average AUROCs. Paired t-test was conducted to compare the performance between DeepScence and each of the other methods. "***" indicates p-value < 0.001, "**" indicates 0.001 < p-value < 0.01, "*" indicates 0.01 < p-value < 0.05, and "NS" indicates not significant (p-value > 0.05). **b,** ROC curves for six example cell types in *in vivo* datasets under standard preprocessing, showing methods with top performance in *in vitro* datasets. **c,** Distribution of SenCID and DeepScence scores under standard preprocessing, comparing cells from healthy and diseased conditions. Wilcoxon test was conducted to compare the two distributions in each case. '*' indicates p-value between 0.01 and 0.05, '**' indicates p-value between 0.001 and 0.01, and '***' indicates p-value < 0.001.
